# Effects of COVID-19 Infection on Spermatogenesis, Oxidative Stress and Erectile Function

**DOI:** 10.3390/jcm12227099

**Published:** 2023-11-15

**Authors:** Peter Törzsök, David Oswald, Christopher Steiner, Michael Abenhardt, Christian Ramesmayer, Ljiljana Milinovic, Bethseba Plank, Zoe Tischleritsch, Lukas Lusuardi, Susanne Deininger

**Affiliations:** 1 Department of Urology and Andrology, Salzburg University Hospital, Paracelsus Medical University, 5020 Salzburg, Austria; d.oswald@salk.at (D.O.); christopher.steiner@gmx.at (C.S.); m.abenhardt@salk.at (M.A.); c.ramesmayer@salk.at (C.R.); l.milinovic@salk.at (L.M.); be.plank@salk.at (B.P.); l.lusuardi@salk.at (L.L.); s.deininger@salk.at (S.D.); 2 Fachhochschule Salzburg, 5431 Salzburg, Austria; zoe.tischleritsch@akhwien.at

**Keywords:** COVID-19, oxidative stress, semen analysis, testosterone, male fertility, virus, infection

## Abstract

Background: Our aim was to evaluate the effect of COVID-19 infection on male fertility and sexual function. Methods: Thirty-one patients were investigated over a mean follow-up of 90 days (22–527) after a COVID-19 infection. Erectile dysfunction (ED), blood tests for sexual hormones, semen analysis including analysis of oxidative stress (OS), as well as COVID-19 antibody titer and the nasal COVID-19 PCR test were evaluated pre- and post-infection. Results: Five patients reported a mild de novo ED (16.13%). One patient had a de novo positive mixed antiglobulin reaction test after the infection. We found no significant difference between pre-COVID-19 and post-COVID-19 spermiogram parameters (*p* = 0.815). OS showed no significant association with COVID-19 infection, but with pathological spermiogram categories, sperm concentration, total sperm count, testis volume, FSH and testosterone. Conclusion: COVID-19 infection does not appear to affect sperm quality and OS negatively in the intermediate term. Further investigations will be needed to assess the potential long-term effects of the infection and vaccination on male sexual function and fertility.

## 1. Introduction

The outbreak of the coronavirus disease (COVID-19) caused by the severe acute respiratory syndrome coronavirus type 2 (SARS-CoV-2) was first reported in 2019 [1]. The angiotensin-converting enzyme 2 (ACE2) protein has been described as the relevant receptor for virus entry, and the transmembrane protease serine 2 (TMPRSS2) is responsible for the priming of the spike protein [2]. COVID-19 may affect several organs with ACE2 expression in the human body, such as the lungs, liver, digestive system, heart, central nervous system, and urinary tract [3]. ACE2 has also been identified in Leydig cells, spermatogonia and Sertoli cells, which would be indicative of a potential effect of SARS-CoV-2 on testicular function [4,5]. Furthermore, COVID-19 may cause neurological symptoms such as dizziness, headache, and impaired sense of taste/smell, which points to the possible penetration of the blood–brain barrier by the virus [6]. The virus itself has been identified in various human secretions, such as saliva, tears, and stool [7]. However, the presence of the virus in the genitourinary system and specifically in semen is controversial [8,9], as the expression of ACE2 and TMPRSS2 in testicular tissue appears to be low [10]. In addition, ejaculate samples would be susceptible to contamination with SARS-CoV-2 during collection [10]. About 7% of the patients reported clinical orchitis due to COVID-19 infection [11].

Several studies demonstrated impaired spermiogenesis after a COVID-19 infection [9,12]. Scrotal hyperthermia due to fever was shown to cause a reversible decrease in spermiogenesis, in which oxidative stress (OS) may play a role [13]. According to Pasqualotto et al., OS is related to male infertility: patients with fertility problems had higher levels of OS compared to a control group [14]. Furthermore, sperm concentration and morphology were negatively correlated with OS [14,15]. However, in the case of COVID-19, the negative effect of fever on sperm quality could not be confirmed. Thus, other mechanisms may affect sperm production in this setting [12]. In vitro studies suggested that the COVID-19 virus disrupts the blood–testis barrier via downregulation of junctional proteins such as occludin, claudin-11 and connexin-43, and may thus impair spermiogenesis [16].

Yet, sperm quality appears to improve over time after recovery from a COVID-19 infection, suggesting a reversible effect on sperm quality [17]. The COVID-19 vaccination, on the other hand, does not seem to affect sperm quality after a follow-up of 6–14 months (for the BNT162b2 vaccine) [18].

In addition to sperm quality, erectile function may be impaired after a COVID-19 infection [19]. Some of the postulated reasons include direct endothelial damage caused by the infection itself, or psychological issues because of the stress due to the pandemic situation [20,21].

The aim of the present study was to investigate sperm quality, including oxidative stress, sexual hormones and sexual function, in a patient population from our clinic who had a confirmed COVID-19 infection and a pre-COVID-19 spermiogram available.

## 2. Materials and Methods

**Figure 1:**
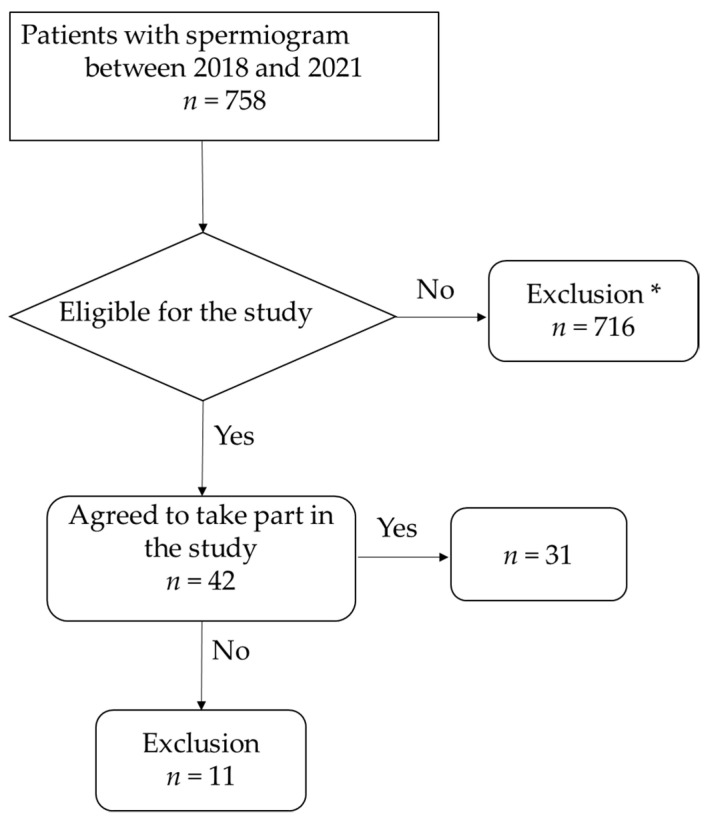
Flowchart of patient selection. * Patients under <18 and >50 years of age, who did not wish to take part in the study or with incomplete data, were excluded from the study.

Patients who had a recorded spermiogram at the University Clinic of Urology and Andrology Salzburg between November 2018 and December 2021 were contacted. Thirty-one patients with a spermiogram prior to a proven COVID-19 infection agreed to participate in the study (Figure 1).

The study was planned to be exploratory. The number of 31 patients available in the study centre was classified as sufficient to enable valuable findings.

The indications for andrological consultation were an unfulfilled desire for a child (*n* = 28), testicular cancer (*n* = 2), or hematospermia (*n* = 1). All participants gave their written informed consent and were >18 years of age at the time of the examinations.

The patients underwent a standardized andrological evaluation including medical history-taking with a questionnaire (covering the issues of family planning, COVID-19 symptoms, COVID-19 vaccinations, body mass index (BMI), smoking history, medication, drug and alcohol consumption, sports, and physical status). The International Index of Erectile Function (IIEF-5) questionnaire was used for the analysis of sexual function. The physical examination included an ultrasound investigation of the scrotum, a blood test, a spermiogram with routine seminal parameters, and determination of OS and antisperm antibodies (ASA). Physical status was rated on a scale from one to five points (one if unfit, five if fit) and whether the patients felt fit or not.

A blood sample was taken between 7 a.m. and 9 a.m. for the investigation of COVID-19 antibodies, FSH, LH, testosterone, free testosterone (fT), sexual hormone binding globulin (SHBG), albumin and prolactin. The normal ranges were as follows: FSH 1.5–12.4 mU/mL; LH 1.7–8.6 mU/mL; testosterone 2.49–8.36 ng/mL; fT 6.76–22.76 pg/mL; SHBG 18.30–54.10 nmol/L; albumin 3.4–5 g/dL; prolactin 86–324 µU/mL. The qualitative analysis of SARS-CoV-2 antinucleocapsid antibodies was performed using SARS-CoV-2 IgG (Abbott, Chicago, IL, USA; Architect i2000SR). Patients were classified according to their qualitative IgG analysis when they had had a COVID-19 infection in the last few months. The quantitative analysis of the SARS-CoV-2 anti-spike antibody was performed using the SARS-CoV-2 IgG II Quant assay (Architect i2000SR, Abbot; unit of measure: binding antibody unit (BAU)/mL). All patients underwent a nasopharyngeal COVID-19 test by polymerase chain reaction (PCR). The SARS-CoV-RNA was analyzed using the Abbott RealTime SARS-CoV-2 assay (ABBOTT^®®®^ Alinity M; Abbott Laboratories, Chicago, IL, USA).

Semen samples were collected in the hospital by masturbation after 2–5 days of sexual restraint. All samples were analyzed within one hour after collection, in accordance with the World Health Organization 2010 (WHO) criteria. Antisperm antibodies (ASA) were analyzed with the mixed antigen reaction test (MAR test). OS was determined by the male infertility oxidative system (MiOXSYS, Aytu BioScience, Inc., Englewood, CO, USA) [22]. The normal value for oxidative stress was <1.38 mV/106 mL.

### Statistics

All data of continuous variables were checked for normal distribution (test of normality: Kolmogorov–Smirnov with Lilliefors significance correction, type I error = 10%).

Pre–post comparisons of continuous variables with normally distributed data were performed by the paired *t*-test. Otherwise, and for comparisons of variables measured on ordinal scales, the exact Wilcoxon test was used. Categorical variables were compared either by the exact McNemar test or by the McNemar–Bowker test.

The impact of the time interval from the last COVID infection to the control investigation, age, the number of vaccinations, COVID antibodies, and specific pre-COVID findings on several spermiogram variables was investigated by multiple linear and logistic regression analysis.

Correlations were reviewed by (point biserial) Bravais–Pearson correlation coefficients and (point biserial) Spearman’s rank correlation coefficients. Associations of continuous variables with categorical variables were investigated by eta² coefficients (combined with a Kruskal–Wallis one-way analysis of variance). Associations of dichotomous variables with categorical variables were investigated by Cramer’s V and Phi coefficients (combined with the exact chi-square test and Fisher’s exact test).

The type I error was not adjusted for multiple testing. Therefore, the results of inferential statistics are only descriptive. Statistical analyses were performed using the open-source R statistical software package, version 4.1.2 (The R Foundation for Statistical Computing, Vienna, Austria). The detailed statistical analysis can be obtained on request from the authors.

## 3. Results

### 3.1. Demographic Data—Descriptive Statistics

Demographic data are summarized in Table 1.

The investigation comprised 31 patients aged on average 35 years (SD 6.8). Eleven patients (35.48%) took some type of medication (Sultanol, Seretide, Levothyroxin, Aspirin, Levocastabine and Frovatriptan) and two patients (6.45%) consumed illegal drugs. Twenty-five patients (80.65%) consumed alcohol regularly. Most of the patients felt fit (*n* = 29/31; 93.55%) and did sports regularly (26/31; 83.87%). The mean testicular volume was 16.45 mL (SD 4.14) on the left side and 18.00 mL (SD 4.76) on the right side.

Two patients had hypothyroidism and were on hormone substitution. No Cushing Syndrome was recorded in the population. In just one case, a Varicocele Gr. I was diagnosed, while two patients had a subclinical varicocele. One patient had a varicocelectomy, one patient had a vaso-vasostomy, one had an orchidopexy as a child, and two patients had a semicastration in case of testicular torsion and testicular malignancy, respectively.

#### 3.1.1. COVID-19: Symptoms, PCR, Virus Variants, Vaccinations, Antibody Titer

**Figure 2:**
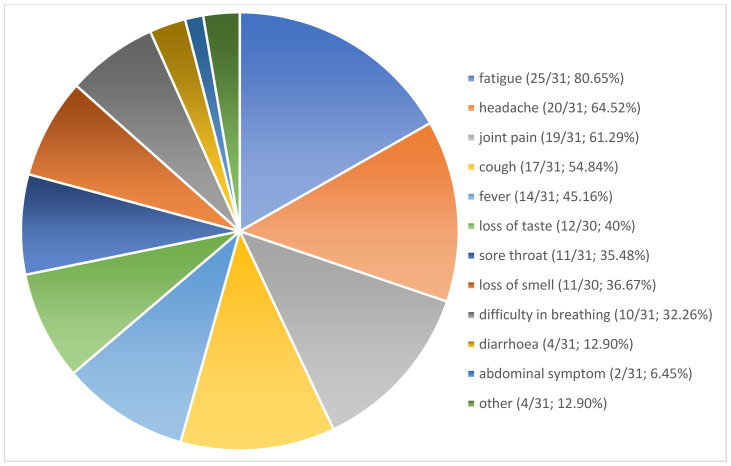
COVID-Infection related symptoms. Other symptoms such as tachycardia, numbness, circulatory issues, sniff (1/31; 3.2%, respectively).

The acute symptoms reported during the COVID-19 infection were fatigue (*n* = 25/31; 80.65%), headache (*n* = 20/31; 64.52%), joint pain (*n* = 19/31; 61.29%), cough (*n* = 17/31; 54.84%), fever (*n* = 14/31; 45.16%), loss of taste (*n* = 12/30; 40%), sore throat (*n* = 11/30; 35.48%), loss of smell (*n* = 11/30; 36.67%), difficulty in breathing (*n* = 10/31; 32.26%), diarrhea (*n* = 4/31; 12.90%), and other symptoms such as tachycardia, numbness, circulatory issues, and sniffing (*n* = 1/31; 3.2%) (Figure 2).

Four patients required medical consultation from a general practitioner due to the infection. No hospitalization was reported. Long-lasting symptoms (long COVID according to the WHO) were reported in 12 cases (38.71%); these included fatigue, shortness of breath, loss of smell/taste, and a burning sensation in the chest [23].

The detected virus variants were the following: wild-type in three patients (9.68%), delta in five patients (16.13%), omicron in 11 patients (35.48%), and a combination of delta and omicron or wild-type and omicron in four patients (12.9%). The virus variant was unknown in eight cases (25.81%) (Table 2).

Three patients had a recurrent infection; two of them had not received any vaccination while the third patient had taken one vaccination.

Six patients received zero, four patients one, nine patients two, and twelve patients three doses of a COVID-19 vaccine. One patient (3.23%) received AstraZeneca (Cambridge, UK), one patient (3.23%) Moderna (Cambridge, MA, USA), 18 patients Pfizer (New York, NY, USA), one patient a combination of Johnson & Johnson (New Brunswick, NJ, USA) and Pfizer, and two patients a combination of AstraZeneca and Pfizer or Moderna and Pfizer vaccine (Table 3).

The COVID PCR test was positive at the time of control in one case (cycle threshold value (CT) 33.88), 22 days after the onset of symptoms. No COVID antibodies were detected in nine patients (29.03%), while the remaining 22 patients (70.97%) had measurable antibody titers. One patient revealed no COVID antibodies within 134 days after the COVID-19 infection and without a vaccination. The mean COVID antibody titer was 1634 BAU/mL (Table 1).

#### 3.1.2. Erectile Function

Twenty-nine patients (93.5%) were satisfied with their sexual lives before the COVID-19 infection. Two patients reported premature ejaculation (6.45%). Five patients had mild erectile dysfunction (16.13%) according to the IIEF-5 questionnaire. De novo premature ejaculation was reported by one patient (3.23%) and reduced libido by five patients (16.13%). After the infection, 29 patients reported unaltered, one patient improved, and one patient worsened sexual function compared to their condition before the disease. Two patients mentioned breathing difficulties during sex and a shorter duration of erection after the COVID-19 infection. No patient registered a disturbance of orgasm, pain during intercourse, or a decreased ejaculate volume.

#### 3.1.3. Laboratory Parameters

Laboratory parameters are summarized in Table 1 and Table 4. One patient at 111 days after the COVID-19 infection had a marginally low testosterone value (2.64 ng/mL) with normal LH and free testosterone levels. All other patients had normal post-COVID-19 testosterone values. Free testosterone, FSH and LH were within the normal range in all post-COVID-19 cases. However, pre-COVID-19 values were available only in six cases. One patient had a pre-COVID-19 testosterone deficiency (2.08 ng/mL) with normal FSH and LH, and a normal post-COVID-19 testosterone level (6.01 ng/mL). The mean OS was 0.66 (SD 8.57); OS was elevated in 10 cases.

The difference between pre- and post-COVID-19 laboratory parameters is summarized in Table 5.

#### 3.1.4. Semen Parameters

Semen parameters are listed in Table 4. Pre-COVID-19 findings revealed normozoospermia in 24 cases (77.42%), while the post-COVID-19 spermiogram indicated normozoospermia in 22 cases (70.96%). The spermiogram was pathological in nine post-COVID-19 and seven pre-COVID-19 cases. Oligozoospermia was seen in no pre-COVID-19 and four post-COVID-19 cases. Two pre-COVID-19 patients and only one post-COVID-19 patient had asthenozoospermia. Oligozoasthenozoospermia was seen in one pre-COVID-19 case and two post-COVID-19 cases. Pyospermia was observed in three pre-COVID-19 patients and one post-COVID-19 patient. Hematospermia was seen in one patient in the pre-COVID-19 and post-COVID-19 cohorts, respectively. The statistical analysis revealed no significant difference between pre-COVID-19 and post-COVID-19 spermiogram findings (McNemar–Bowker test; *p* = 0.815).

Two patients were initially positive on the MAR test (6.45%): one had undergone vasovasostomy and one had mumps orchitis in his history. After a COVID-19 infection, a third patient with oligozoospermia was MAR positive. The bacteriology was positive in six patients pre-COVID-19 and in four patients post-COVID-19 (19.35% and 12.90%, respectively).

The post-COVID-19 spermiogram revealed significantly better fast progressive motility (Grade A) when compared to pre-COVID-19 values (*p* = 0.005; *t*-test). The post-COVID-19 semen volume was lower but not significantly so (*p* = 0.078, Wilcoxon). None of the other investigated post-COVID-19 semen parameters were significantly different from pre-COVID-19 values.

#### 3.1.5. Correlation and Regression Analysis

The time from the COVID infection to the control investigation showed a significant negative correlation with COVID-19 antibody titers (−0.383; *p* = 0.037; Spearman).

The qualitative COVID-19 antibody investigation showed a significant correlation with grade b motility (0.410; *p* = 0.022), no motility (−0.366; *p* = 0.043), and the time from COVID-19 infection to the control investigation (−0.383; *p* = 0.037). The quantitative COVID-19 antibody examination was significantly correlated with the absolute and relative difference in semen volume (−0.422, *p* = 0.013; −0.442, 0.013), and with the absolute difference in fast progressive and slow progressive sperm motility (−0.411, *p* = 0.022; 0.358, *p* = 0.048). A detailed correlation analysis of OS and various parameters is shown in Table 6.

The impact of the time from the last COVID-19 infection to the control investigation, age, COVID-19 antibody, and the specific pre-COVID-19 findings on several spermiogram variables investigated by multiple linear and logistic regression analyses revealed a significant impact on post-COVID-19 morphology only for age (*p* = 0.017; regression co-efficient −4.217). The detailed regression and correlation analyses can be obtained on request from the authors.

## 4. Discussion

Since the outbreak of the COVID-19 pandemic in 2019, millions of individuals have contracted the virus. The urological effects of the infection are controversially discussed. The impact of a viral infection on semen quality has been reported in detail for mumps, Zika, Ebola, and the human immunodeficiency virus (HIV) [24]. Male sexual hormones are the central factor involved in male sexual function and erection. Normal healthy functioning of this system requires a complex interplay of the nervous system, hormonal glands and the target organ. The exact role of a COVID-19 infection in this interplay remains unclear, although COVID-19 may undoubtedly affect the pituitary gland [25].

A meta-analysis of 17 studies in 2023, comprising more than 1600 patients with a former COVID-19 infection, showed no evidence of decreased FSH, LH or testosterone after recovery. Nevertheless, there seemed to be an effect in another hormonal axis: estradiol (E2) (*p* = 0.001) and prolactin (*p* = 0.022) were significantly increased after the infection [10]. On the other hand, a study of 74 male patients after COVID-19 infection with a mean follow-up of 80 days (IQR 64–93) showed normal semen parameters and normal FSH, LH, estradiol and testosterone values. However, when compared to controls, the sperm concentration, total sperm count and total motility were significantly lower in the post-infection group [9].

Our study confirmed these findings: all post-COVID-19 testosterone levels were within the normal range, but pre-COVID-19 testosterone values were available only for six cases. In a meta-analysis, Wang et al. lacked sufficient data and were therefore unable to perform a subgroup analysis of the effect of COVID-19 on male sexual hormones divided by the severity of infection [10]. However, we have data from other research groups which show that a particularly serious COVID-19 illness may well lead to the disruption of male sexual hormones. According to a study comprising 384 patients, a COVID-19 infection, especially with pulmonary involvement, may result in decreased testosterone and elevated LH compared to pre-COVID-19 values (*p* < 0.001) [23]. An investigation of 63 Asian men within 2–14 days after the onset of COVID-19 symptoms showed normal testosterone values, while 27% of the patients had higher LH, and 10% had higher FSH values [26]. A further study investigated the expression of LH, FSH, testosterone and prolactin in patients hospitalized due to COVID-19 (*n* = 89) compared to patients hospitalized with non-COVID respiratory infections (*n* = 30) and a control group (*n* = 143) [25]. The COVID group had significantly lower testosterone levels compared to the other groups (*p* < 0.01). LH and prolactin were significantly higher in hospitalized patients compared to controls, but not between the hospitalized COVID-19 and the hospitalized non-COVID cases. FSH did not reveal a significant difference between the three groups. The polymorphism of the androgen receptor has been studied as another possible cause of a serious COVID-19 infection, as well [27].

Interestingly, a 7-month cohort study demonstrated reduced testosterone levels mainly in patients with more numerous comorbidities, supporting the idea of testosterone being a marker of severity in patients with reduced health status [28]. Over 50% of men who had recovered from a COVID-19 infection still had decreased testosterone values, indicating hypogonadism after a 7-month follow-up [28]. Our population did not include patients with a severe clinical course, which could be an explanation for the normal testosterone values.

Furthermore, our study showed that COVID-19 infection had no negative impact on spermiogram parameters. However, the meta-analysis by Wang et al. came to a different conclusion: here a COVID-19 infection reduced the total sperm count (*p* = 0.012), sperm concentration (*p* = 0.001), total motility (*p* = 0.001), progressive motility (*p* = 0.048), and the viability of sperms (*p* = 0.031) [10]. However, the follow-up time points remain unclear in the meta-analysis of Wang et al. In our study, the mean follow-up period was 90 days, but the maximum follow-up time point was >500 days post-COVID-19 infection. Anti-spike 1 protein and anti-S1-RBD serum IgG antibody titers, according to Donders et al. [12], are negatively correlated with sperm cell count (*p* = 0.008) and motility (*p* = 0.04). Within a period of one month after a COVID-19 infection, patients had reduced sperm concentrations, total sperm count and motility compared to patients > 1 month after the COVID-19 infection [12]. It is apparent that spermiogram parameters negatively influenced by COVID-19 may recover over time. We found no significant correlation between the time from COVID-19 infection to the control investigation in regard to the studied parameters, except for the COVID-19 antibody titer (−0.838; *p* = 0.037).

An increasingly important parameter in the examination of the ejaculate is OS, which is associated with major semen parameters, such as sperm concentration, total sperm count, and total and progressive motility [14,15]. These data were confirmed in our cohort: a significant negative correlation was noted between OS and sperm concentration, total sperm count, progressive motility, fast progressive motility and testis volume. As regards testosterone, FSH, volume of the testis and spermiogram category, we noted a significant positive correlation in our cohort. OS is one of the main causes of deoxyribonucleic acid (DNA) fragmentation, and thus of impaired sperm quality [29]. DNA fragmentation may be increased by febrile infections: In 2000, Evenson et al. observed an increase in DNA fragmentation due to fever and presumably elevated temperature in the testis of a patient with influenza [30]. In 2022, Shcherbitskaia et al. registered a correlation for COVID-19: the DNA fragmentation rate was negatively correlated with the duration of recovery from the infection [31]. In a subgroup analysis, Wang et al. [10] also found that patients with a febrile COVID-19 infection had significantly lower sperm concentrations (*p* = 0.02) and a lower progressively motile sperm count (*p* = 0.01) compared to individuals without fever. COVID-19 may cause inflammatory cell infiltration in the testes [10]. Thus, indirect damage to spermiogenesis by fever and possibly immunogenic factors appears much more likely than damage by the virus itself.

However, our study does not permit a clear distinction between the negative effect of the infection itself and the vaccination on male sexual hormones and spermiogram parameters. Only six patients had received no vaccination prior to the post-COVID-19 spermiogram. All others had taken different numbers of different vaccines.

According to a cell culture study with bovine anterior pituitary cells, the application of increasing concentrations of the recombinant spike protein of SARS-CoV-2 results in the suppression of LH and FSH secretion. However, the suppression mechanism of FSH was seen in the case of higher spike-protein concentrations [32].

A minimum follow-up of 74 days (the maturation time of spermatozoa) should be considered when evaluating the long-term effects of COVID-19 and vaccination on sperm quality. This would rule out misinformation to patients recovering from an infection. According to a recent meta-analysis comprising 29 studies from October 2022 onward, there is actually no scientific evidence of COVID-19 vaccines exerting a negative impact on male or female fertility [33].

High levels of ASA have been reported in about 2.5% of COVID-19 patients [12]. An association between viral infections and ASA has been registered for other viral infections as well (human papillomavirus (HPV) [34]. In our population, one patient was de novo MAR-positive after the COVID-19 infection (3.2%), which could be due to an orchitis caused by the virus itself. This is a cause of concern and should be investigated over a longer follow-up period in larger patient numbers. There are some reports on the possible effect of a COVID-19 infection on the testis concerning orchitis [11]. Furthermore, testicular damage due to a COVID-19 infection can be caused by an inflammatory reaction and OS [35].

According to a study comprising 81 patients with mild to moderate ED, post-COVID-19, IIEF-5 values were significantly deteriorated compared to pre-COVID-19 values in both groups [19]. In our population, one patient reported a poorer sexual life after COVID-19, one patient reported de novo premature ejaculation, and two patients had breathing difficulties during intercourse. Yet, erection and sexual function are multifactorial phenomena: the endothelial dysfunction described in a COVID-19 infection, possible pudendal nerve damage, together with immune-inflammatory factors, could serve as an explanation for a potential post-COVID-19 ED at the molecular level. However, psychological factors such as the threat of loss of employment, social isolation or other events could contribute to ED [21].

The body of data regarding male sexuality and fertility during and after COVID-19 infection is heterogeneous. Especially in combination with the potential effects of an additional vaccination, the long-term effects are not foreseeable. Many people have experienced a COVID-19 infection: on 18 August 2023, there were nearly 770 million confirmed cases of COVID-19 infection worldwide, with a large number of unreported cases. This warrants a wait-and-watch approach in regard to a potential impairment of male health in the future [36].

The limitations of the present study are that pre-COVID-19 laboratory parameters were only available in a few cases, and the sample size was relatively small. Given the different follow-up periods, the immediate short-term effect of an acute COVID-19 infection on sperm parameters cannot be ruled out. Furthermore, due to the heterogeneity of the vaccinations, a reliable analysis of the effect of the vaccinations was not possible.

## 5. Conclusions

A COVID-19 infection does not appear to exert a negative effect on sperm quality and oxidative stress in a follow-up period of 90 days (22–527) after the onset of symptoms. ASA and de novo erectile dysfunction after COVID-19 infection should be a cause of concern and warrant further investigation.

## Figures and Tables

**Table 1:** Patients’ clinical data and laboratory parameters.

	Median	IQR	Min.	Max.	*n*
Time from the COVID-19 infection to the control investigation (days)	90	70	22	527	30
Time between spermiograms (days) ^a^	594	787	228	1389	31
Age (years)	35	11	22	49	31
Height (m)	1.8	0.1	1.63	1.9	31
Weight (kg)	80	12	61	105	31
BMI (kg/m^2^) ^b^	24.39	3.59	20.45	32.19	31
Pack years	7	7	4	30	5
Physical status scale (1–5; 1 unfit, 5 fit)	4	1	2	5	31
IIEF-5 ^c^	24	2	18	25	31
MiOXSYS (mV/10^6^)—oxidative stress	0.66	2.67	0.13	32.95	31
COVID-19 antibody (BAU/mL)	1634	7671	<7	11,360	31

^a^ Time period between the first spermiogram and the control spermiogram performed for the study; ^b^ BMI body mass index; ^c^ International Index of Erectile Function; IQR: Interquartile range.

**Table 2:** Virus variants.

Virus Variant	*n*	%
Unknown	8	25.81
wild-type (W)	3	9.68
Delta (D)	5	16.13
Omicron (O)	11	35.48
Combination: D + O or W + O	4	12.9
Total	31	100

**Table 3:** Type of Vaccinations.

Vaccine	*n*	%
none	6	19.35
AstraZeneca ^a^	1	3.23
Pfizer ^b^	18	58.06
Moderna ^c^	1	3.23
AstraZeneca + Pfizer	2	6.45
Moderna + Pfizer	2	6.45
Johnson&Johnson ^d^ + Pfizer	1	3.23
Total	31	100

Six patients received no, four patient one, nine patient two and 12 patient three vaccinations. ^a^ Cambridge, UK; ^b^ New York, NY, USA; ^c^ Cambridge, MA, USA; ^d^ New Brunswick, NJ, USA.

**Table 4:** Semen and laboratory parameters.

		Pre-COVID					Post-COVID			
	Median	IQR	Min.	Max.	*n*	Median	IQR	Min.	Max.	*n*
Volume (mL)	4.4	2.2	1.8	12	31	3.9	2.1	1.2	10	31
pH	7.5	0.6	6.7	8	31	7.7	0.3	7	8	31
Sperm concentration (million/mL)	51.2	54.6	1	252.8	31	61.6	80.2	2	208	31
Total sperm count (million)	167	145.5	5.2	921.6	31	235.5	167.6	6.8	688	31
Vitality (%)	71	12	20	88	31	72	18	40	89	31
Progressive motility (a + b) (%)	49	16	18	70	31	52	17	15	85	31
Fast progressive motility a (%)	18	18	0	49	31	25	20	5	45	31
Slow progressive motility b (%)	30	18	16	54	31	25	24	5	47	31
Non-progressive motility c (%)	12	6	2	26	31	11	12	2	20	31
No motility d (%)	38	13	20	60	31	38	22	10	82	31
Morphology (%)	11	9	3	40	31	10	12	3	32	31
Round cells (million/mL)	0.8	1	0.4	8.8	31	0.8	2	0.4	16.1	31
Leukocytes (million/mL)	0	0.4	0	4.9	31	0	0.2	0	9.1	31
Testosterone (ng/mL)	4.47	2.63	2.08	5.79	6	4.85	2.15	2.64	8.05	31
Free testosterone (pg/mL)	15.4		15.4	15.4	1	11.82	3.72	4.82	26.98	31
FSH ^a^ (mU/mL)	6.1	3.3	3.2	9.4	6	4.4	4	1.6	10.9	31
LH ^b^ (mU/mL)	5.1	3	2.1	5.6	6	4.2	2.4	2	7.2	31
Prolactin (µU/mL)	190	154	72.8	357	6	205	129	15.3	475	31
SHBG ^c^ (nmol/L)	29.6		29.6	29.6	1	34.1	20.5	12.4	74.9	31

^a^ follicle-stimulating hormone; ^b^ luteinizing hormone; ^c^ sexual hormone binding globulin; IQR: Interquartile range.

**Table 5:** Absolute and relative difference between post-COVID-19 and pre-COVID-19 laboratory parameters.

		Absolute Difference					Difference in%			
Parameter	Median	IQR	Min.	Max.	*n*	Median	IQR	Min.	Max.	*n*
Semen volume (mL)	−0.50	1.7	−4	4.8	31	−11.11	47.62	−50	100	31
pH	0.10	0.4	−0.8	0.6	31	1.32	5.45	−10	8.33	31
Sperm concentration (million/mL)	1.40	25.8	−191.2	132.4	31	7.47	105.13	−78.79	186.67	31
Total sperm count (million)	1.60	175.1	−712.3	544	31	17.00	100.89	−85.3	377.78	31
Vitality (%)	−2.00	16	−30	24	31	−2.86	23.78	−42.86	105	31
Progressive motility (a + b)	4.00	23	−28	37	31	7.14	48.14	−55.1	161.11	31
Fast progressive motility (a)	5.00	21	−12	34	31	19.05	150.83	−63.16	1100	29
Slow progressive motility (b)	1.00	22	−37	23	31	5.56	90.3	−85	95.83	31
Non-progressive motility (c)	0.00	14	−18	16	31	0	86.36	−85.71	533.33	31
No motility (d)	−4.00	25	−28	28	31	−15.00	61,33	−73.68	120	31
Morphology (%)	1.00	9	−13	18	31	10.00	58.04	−65	233.33	31
Round cells (million/mL)	0.00	1.2	−6.4	7.4	31	0.00	132.95	−88.89	925	31
Leukocytes (million/mL)	0.00	0.2	−3.8	4.2	31	−100.00	5	−100	85.71	31
Testosterone (ng/mL)	−0.40	3.79	−2.87	3.93	6	−6.69	112,5	−49.57	188.94	6
FSH (mU/mL)	0.45	3.1	−0.7	2.9	6	6.25	42.63	−7.45	56	6
LH (mU/mL)	0.60	1.7	−1.4	1.5	6	14.64	59.69	−25	52.38	6
Prolactin (µU/mL)	50.00	173	−177	116.2	5	34.97	76.46	−49.58	159.62	5

To determine the difference between pre-COVID-19 and post-COVID-19 values, the latter were subtracted from the former. The difference in percentage was calculated by subtracting the post-COVID-19 percentage from the pre-COVID-19 value (pre-COVID-19 = 100%). As a further note on the calculation of differences, positive values implied an increase in post-COVID-19 values compared to pre-COVID-19 values, whereas negative values signified a decrease in post-COVID-19 values compared to pre-COVID-19 values. IQR: Interquartile range.

**Table 6:** Correlations analyses of oxidative stress with the investigated parameters.

Investigated Parameters	Correlations Coefficient	*p*-Value
Sexual abstinence (days)	−0.299	1.03
Semen volume (mL)	0.248	0.179
Semen pH	0.116	0.534
**Sperm concentration (million/mL)**	**−0.482**	**<0.001**
**Total sperm count (million)**	**−0.783**	**<0.001**
Vitality (%)	−0.244	0.186
**Progressive motility (a + b%)**	**−0.357**	**0.049**
**Fast progressive motility (a%)**	**−0.489**	**0.005**
Slow progressive motility (b%)	−0.054	0.771
Non-progressive motility (c;%)	0.14	0.452
No motility (d%)	0.284	0.121
Morphology (%)	0.126	0.499
**Round cell count (million/mL)**	**−0.355**	**0.04992**
Leukocytes (million/mL)	−0.13	0.486
**Testosterone (ng/mL)**	**0.4**	**0.026**
Free testosterone /pg/mL)	−0.35	0.853
**FSH (mU/mL)**	**0.38**	**0.035**
LH (mU/mL)	−0.056	0.763
Prolactin (µU/mL)	0.211	0.254
SHBG (nmol/L)	−0.21	0.257
Agglutination	−0.354	0.051
MAR test	0.122	0.538
Bacteriology (positive/negative)	−0.194	0.297
COVID-PCR at control	−0.214	0.247
Time from the last COVID infection to the control investigation	0.183	0.333
Time between spermiograms	0.271	0.14
Symptom duration of COVID infection (days)	−0.076	0.683
Age (years)	−0.065	0.728
Height (m)	0.037	0.844
Weight (kg)	0.005	0.977
BMI (kg/m^2^)	0.117	0.532
**Testis volume left (mL)**	**−0.394**	**0.031**
**Testis volume right (mL)**	**−0.494**	**0.007**
IIEF-5—erectile function	−0.076	0.686
COVID antibody titer (BAU/mL)	0.114	0.543
COVID antibody qualitative	−0.286	0.119
**Spermiogram category (normal vs. non-normal)**	**0.473**	**0.007**
Alcohol consumption (yes/no)	−0.292	0.111
Illegal drug consumption (yes/no)	0.162	0.385
Smoking (yes/no)	−0.231	0.211
Medication (yes/no)	0.011	0.952
Number of COVID infections	0.069	0.714
Cough (yes/no)	−0.94	0.614
Fever (yes/no)	0.033	0.862
Joint pain (yes/no)	0.13	0.487
Headache (yes/no)	0.083	0.657
Shortness of breath (yes/no)	0.332	0.068
Sore throat (yes/no)	−0.083	0.657
Diarrhea (yes/no)	0.14	0.453
**Loss of smell (yes/no)**	**0.364**	**0.048**
Loss of taste (yes/no)	0.299	0.109
Abdominal symptoms (yes/no)	0.117	0.529
Fatigue (yes/no)	0.008	0.965
Premature ejaculation (yes/no)	−0.82	0.662
Reduced libido (yes/no)	0.029	0.875
Need to see a doctor	0.145	0.436
Long COVID	−0.015	0.937
Fitness scale (1 to 5; 5 is the best)	0.085	0.649
Fit (yes/no)	−0.015	0.938
Active in sports (yes/no)	0.157	0.399
Testis volume is normal (yes/no)	−0.22	0.235

Spearman’s rank correlation or Spearman’s point-biserial rank correlation was used for statistical analysis. Significant changes are marked bold.

## Data Availability

Can be obtained on request from the authors.

## Abbreviations

ACE2	angiotensin-converting enzyme 2
ASA	anti-sperm antibodies
BAU	binding antibody unit
BMI	body mass index
COVID-19	Coronavirus disease
CT	cycle threshold value
DNA	deoxyribonucleic acid
FSH	follicle-stimulating hormone
fT	free testosterone
HIV	human immunodeficiency virus
HPV	human papillomavirus
IQR	interquartile range
LH	luteinizing hormone
MAR test	mixed antigen reaction test
MiOXSYS	Male infertility Oxidative System
OS	oxidative stress
PCR	polymerase chain reaction
ROS	reactive oxygen species
SARS-CoV-2	severe acute respiratory syndrome coronavirus-2
SD	standard deviation
St.p.	status post (an event a patient has experienced prior to the current time)
TMPRSS2	transmembrane protease serine 2
WHO	World Health Organisation
